# Increasing the accuracy of single sequence prediction methods using a deep semi-supervised learning framework

**DOI:** 10.1093/bioinformatics/btab491

**Published:** 2021-07-02

**Authors:** Lewis Moffat, David T Jones

**Affiliations:** Department of Computer Science, University College London, London WC1E 6BT, UK; Biomedical Data Science Laboratory, The Francis Crick Institute, London NW1 1AT, UK; Department of Computer Science, University College London, London WC1E 6BT, UK; Biomedical Data Science Laboratory, The Francis Crick Institute, London NW1 1AT, UK

## Abstract

**Motivation:**

Over the past 50 years, our ability to model protein sequences with evolutionary information has progressed in leaps and bounds. However, even with the latest deep learning methods, the modelling of a critically important class of proteins, single orphan sequences, remains unsolved.

**Results:**

By taking a bioinformatics approach to semi-supervised machine learning, we develop Profile Augmentation of Single Sequences (PASS), a simple but powerful framework for building accurate single-sequence methods. To demonstrate the effectiveness of PASS we apply it to the mature field of secondary structure prediction. In doing so we develop S4PRED, the successor to the open-source PSIPRED-Single method, which achieves an unprecedented *Q*_3_ score of 75.3% on the standard CB513 test. PASS provides a blueprint for the development of a new generation of predictive methods, advancing our ability to model individual protein sequences.

**Availability and implementation:**

The S4PRED model is available as open source software on the PSIPRED GitHub repository (https://github.com/psipred/s4pred), along with documentation. It will also be provided as a part of the PSIPRED web service (http://bioinf.cs.ucl.ac.uk/psipred/).

**Supplementary information:**

Supplementary data are available at *Bioinformatics* online.

## 1 Introduction

Over the past two decades, sequence-based bioinformatics has made leaps and bounds towards better understanding the intricacies of DNA, RNA and proteins. Large sequence databases (UniProt-Consortium, 2019) have facilitated especially powerful modelling techniques that use homology information for a given query sequence to infer aspects of its function and structure (Kandathil *et al.*, 2019b). A keen example of this progress is in current methods for protein structure prediction that utilize multiple sequence alignments (MSAs) and deep learning to accurately infer secondary and tertiary structure (Greener *et al.*, 2019; Jones, 2019; Senior *et al.*, 2020). Unfortunately, much of this progress has not extended to orphan sequences, a very important but very difficult to model class of sequences which have few to no known homologous sequences (Greener *et al.*, 2019; Levitt, 2009; Perdigão *et al.*, 2015). Also, even when homologues are available, multiple sequence alignment is often too slow to apply to the entirety of a large sequence data bank, and so improved annotation tools which can work with just a single input sequence are also vital in maintaining resources such as InterPro (Blum *et al.*, 2021).

Here, we present Profile Augmentation of Single Sequences (PASS), a general framework for mapping multiple sequence information to cases where rapid and accurate predictions are required for orphan sequences. This simple but powerful framework draws inspiration from Semi-Supervised Learning (SSL) to enable the creation of massive single-sequence datasets in a way that is biologically intelligent and conceptually simple. SSL methods represent powerful approaches for developing models that utilize both labelled and unlabelled data. Where some recent works (Alley *et al.*, 2019; Heinzinger *et al.*, 2019) have looked to take advantage of unlabelled biological sequence data using unsupervised learning, borrowing from techniques in natural language processing (Dai *et al.*, 2019; Devlin *et al.*, 2019), we instead look to modern SSL methods like FixMatch (Sohn *et al.*, 2020) for inspiration. These methods have demonstrated that pseudo-labelling, amongst other techniques, can significantly improve model performance (Berthelot *et al.*, 2019; Lee, 2013; Sohn *et al.*, 2020). Pseudo-labelling techniques use the model being trained to assign artificial labels to unlabelled data, which is then incorporated into further training of the model itself (Lee, 2013).

**Fig. 1.**
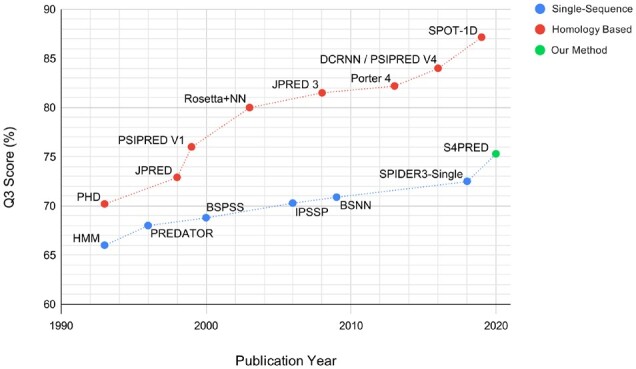
Plot showing reported test *Q*_3_ scores for a range of published secondary structure prediction methods over the previous three decades. This includes single-sequence methods (Asai *et al.*, 1993; Aydin *et al.*, 2006; Bidargaddi *et al.*, 2009; Frishman and Argos, 1996; Heffernan *et al.*, 2018; Schmidler *et al.*, 2000) and homology methods (Cole *et al.*, 2008; Cuff *et al.*, 1998; Hanson *et al.*, 2019; Jones, 1999; Li and Yu, 2016; Meiler and Baker, 2003; Mirabello and Pollastri, 2013; Rost and Sander, 1993) separately to provide an illustrative view of how single-sequence methods have improved very slowly, compared to homology methods, over time. We include this work, S4PRED, to demonstrate how it is a step upwards in accuracy. In order to avoid conflation with Rosetta *ab initio*, we use the name Rosetta + Neural Network (Rosetta+NN) in this figure to refer to the work of Meiler & Baker (Meiler and Baker, 2003)

PASS uses a bioinformatics-based approach to pseudo-labelling to develop a dataset for a given prediction task before training a predictive single-sequence model. First, a large database of sequences is clustered into MSAs. Each MSA is then used as input to an accurate homology-based predictor. The predictions are then treated as pseudo-labels for a single sequence from the MSA. This allows a large unlabelled set of single sequences to be converted into a training set with biologically plausible labels, that can be combined with real labelled data, for training a deep learning-based predictor. As an exemplar of the effectiveness of the PASS framework, we apply it to the well explored field of single-sequence secondary structure prediction resulting in Single-Sequence Secondary Structure PREDictor (S4PRED), the next iteration of PSIPRED-Single, our current method. S4PRED achieves a state-of-the-art *Q*_3_ score of 75.3% on the standard CB513 test set (Cuff and Barton, 1999). This performance approaches the first version of the homology-based PSIPRED (Jones, 1999) and represents a leap in performance for single-sequence-based methods in secondary structure prediction (Fig. 1).

Starting from a three class accuracy ($Q_{3}$) of ∼76% (Jones, 1999) in the late 1990s, our secondary structure prediction tool, PSIPRED, has grown to a current state-of-the-art *Q*_3_ of 84.2%, and is used globally in both experimental and computational research (Buchan and Jones, 2019). PSIPRED, along with other methods, is able to produce high accuracy predictions by leveraging valuable homology information found in MSAs (Yang *et al.*, 2018). This approach is in stark contrast to single-sequence methods, like PSIPRED-Single (Buchan and Jones, 2019), that are designed to predict secondary structure based only on a single query sequence, without relying on homology information. Unfortunately, over the past decades, single-sequence methods have been slow to improve relative to homology-based methods, as can be seen in Figure 1. Currently, the most performant single-sequence methods achieve low *Q*_3_ scores of 71–72% (Bidargaddi *et al.*, 2009; Buchan and Jones, 2019; Heffernan *et al.*, 2018; Torrisi *et al.*, 2019), where homology-based methods are achieving scores of >84% (Buchan and Jones, 2019; Hanson *et al.*, 2019; Torrisi *et al.*, 2019) and are approaching a hypothesized theoretical maximum of 88–90% (Rost, 2001).

Accurate single-sequence prediction enables the modelling of any given sequence without the constraints of homology, which represents a valuable research prospect with a plethora of use cases. The most apparent of these is being able to better model any part of the known protein space, especially given that a quarter of sequenced natural proteins are estimated to have no known homologues (Levitt, 2009) and an even larger portion are inaccessible to homology modelling (Greener *et al.*, 2019; Ovchinnikov *et al.*, 2017; Perdigão *et al.*, 2015). For example, a particularly important area where this is often the case is viral sequence analysis. The structures of viral proteins are often attractive targets for the development of antiviral drugs or the development of vaccines (Mokili *et al.*, 2012), however, viral sequences tend to be highly diverse and typically have no detectable homologues, making structural modelling difficult (Edwards and Rohwer, 2005; Mokili *et al.*, 2012; Riesselman *et al.*, 2018). Another example is being able to better model the homology-poor ‘dark proteome’ (Perdigão *et al.*, 2015). The value of single-sequence methods also extends outside of natural proteins to areas like *de novo* protein design (Marcos and Silva, 2018), where novel sequences and structures typically, by their very design, have no homologues (Koga *et al.*, 2012).

Even in the case of a sequence having known homologues, single-sequence methods have many valuable uses. One clear example is in variant effects (Riesselman *et al.*, 2018), where methods like PSIPRED that use MSAs are limited because their predictions for a given sequence will be biased towards a family ‘average’ (Kandathil *et al.*, 2019b). Single-sequence methods avoid this bias in not utilizing any homology information and may have the potential to better model the changes in secondary structure across a family even for highly divergent members. This also extends to being able to better model large single-species insertions that intrinsically have no homology information. Being able to avoid the bias of homology methods could also benefit protein engineering tasks (Yang *et al.*, 2019), where the aim may be to generate a sequence that is highly divergent from its homologues.

## 2 Materials and methods

For S4PRED, we use the PASS framework to develop a pseudo-labelling approach that is used to generate a large set of single sequences with highly accurate artificial labels. The first step is taking a large set of unlabelled protein sequences clustered as alignments and then removing the clusters containing a small number of sequences. The MSA-based PSIPRED V4 (Buchan and Jones, 2019) is then used to generate secondary structure predictions for each remaining cluster alignment. The representative sequence for each cluster is used as the target sequence when predicting secondary structure. The target sequence is then kept along with the three-class predictions, and the alignment is discarded. In this way, each cluster produces a single training example, constituting a single sequence and its pseudo-labels.

This approach effectively utilizes a homology-based predictor to provide accurate pseudo-labels for individual unlabelled sequences. PSIPRED generates high accuracy predictions, so it can be inferred that it is providing highly plausible secondary structure labels. These labels are, therefore, able to provide valuable biological information to the S4PRED model during training. Because each sequence is sampled from a separate cluster, there is also the added benefit of diversity between individual sequences in the dataset.

Training sets are used by the machine learning model to learn the predictive mapping of an amino acid sequence to secondary structure sequence. During training the validation set is used as a means of monitoring the performance of a model, but it does not learn from this set. The test set is the final unseen benchmark set that the trained model is tested against.

In this work, we use the Uniclust30 database (Mirdita *et al.*, 2017) to generate a pseudo-labelled training set, which, after a rigorous process of benchmarking and cross-validation, contains 1.08M sequences with pseudo-labels. To accompany the pseudo-labelled sequences, we construct a labelled training set and a labelled validation set from protein structures in the PDB (Burley *et al.*, 2019). For proper cross-validation, sequences in both the labelled training and labelled validation sets were removed if they were homologous to any sequences in the CB513 test set, evaluated by CATH (Sillitoe *et al.*, 2019) Superfamily-level classification. The final labelled training and validation sets contain 10143 and 534 sequences respectively.

In summary, there is a labelled training set along with a labelled validation set and labelled test set. There is also the pseudo-labelled training set. The neural network model learns from both labelled and pseudo-labelled training sets, and, during training in both cases, the labelled validation set is used to measure overtraining and perform early stopping. The final trained model that has learned from both training sets is then tested against the labelled test set (CB513).

To train the S4PRED model using both sets of data we adapt the ‘fine-tuning’ approach from recent work of Devlin and collaborators (Devlin *et al.*, 2019). In the context of S4PRED, fine-tuning consists of first training on the large pseudo-labelled training set (See Supplementary Material S3), after which a small amount of additional training is performed with the labelled dataset (See Supplementary Material S4). Fine-tuning in this manner provides an effective and regimented training scheme that incorporates both sets of sequences. The S4PRED model itself uses a variant of the powerful AWD-LSTM (Merity *et al.*, 2018) model, a recurrent neural network model that uses a variety of regularization techniques. See Supplementary Figure S2 for a diagram of the neural network model during inference.

### 2.1 Labelled dataset construction

The first stage in our construction of a labelled dataset is generating a non-redundant set of PDB chains using the PISCES server (Wang and Dunbrack, 2003) with a maximum identity between structures of 70% and a maximum resolution of 2.6_** Å**_. This produces a list of 30630 chains, all with a length of 40 residues or more. At the cost of introducing some noise but retaining more examples, we do not remove any chains with unlabelled residues.

From this list, we then remove any chains that share homology with the test set. We use the standard test set for secondary structure prediction, CB513. Homology is assessed and qualified as having any overlapping CATH (Sillitoe *et al.*, 2019) domains at the Superfamily level with any of the sequences in the test set (Jones, 2019). This removes approximately 2/3 of the chains leaving a total of 10677 from which to generate training and validation sets. This approach ensures no test set data leakage in either the labelled training set or the labelled validation se*t*.

The remaining chains are clustered at 25% identity using MMseqs2 (Steinegger and Söding, 2017). From the resulting 6369 clusters, a subset is randomly sampled such that the total sum of their sequences makes up ∼5% of the 10677 chains. This is to create a validation set that achieves a 95%/5% split between training and validation sets, as well as keeping the validation and test sets similarly sized. This leaves a final split of 10143/534/513 examples for the training, validation and test sets respectively.

Secondary structures are specified using DSSP (Kabsch and Sander, 1983). For each residue in each sequence, the eight states (H, I, G, E, B, S, T, –) are converted to the standard 3 classes (*Q*_3_) of strand for E & B, helix for H & G and loop (coil) for the remainder. Protein sequences are represented as a sequence of amino acids, where each residue is represented by one of 21 integers; twenty for the canonical amino acids and one for ‘X’ corresponding to unknown and non-canonical amino acids. Each integer represents an index to a 128-dimensional embedding that is learned by the neural network model during training (See Supplementary Materials S2 and S3 for further architecture details).

### 2.2 Pseudo-labelled dataset generation

To assemble a dataset of pseudo-labelled sequences we start with Uniclust30 (January 2020 release) (Mirdita *et al.*, 2017). This consists of UniProtKB (UniProt-Consortium, 2019) sequences clustered to 30% identity, making up 23.8M clusters. Each cluster is then considered as a single potential example for the pseudo-labelled training set. Any cluster can be converted into a target sequence and alignment which can then be passed to PSIPRED to generate high accuracy predictions of secondary structure. These secondary structure predictions are then one-hot encoded and treated as pseudo-labels with the target sequence providing a single example.

Clusters are filtered from the initial 23.8M Uniclust30 set by removing clusters that are either too short or have too few sequence members. If a cluster has a representative sequence with a length of less than 20 residues or contains less than 10 non-redundant sequences in its alignment it is removed. Applying these restrictions leaves a much smaller set of 1.41M clusters. These are the candidate clusters for generating a training set from which homology with the validation and test sets is to be removed.

### 2.3 Removal of test set homology from the pseudo-labelled dataset

The S4PRED model is trained on labelled and pseudo-labelled data and, as such, the pseudo-labelled set requires removal of sequences homologous to the CB513 (Cuff and Barton, 1999) test set. When S4PRED is training on the pseudo-labelled set it uses the real-labelled validation set for early stopping. As such, we also seek to remove sequences from the pseudo-labelled set that are clearly homologous with the validation set.

For the vast majority of clusters, solved structures are not available. This leaves sequence-based approaches to identify and eliminate clusters that share any homology with the test set. It is widely known that using a simple percent identity (e.g. 30%) as a homology threshold between two sequences is inadequate and leads to data leakage (Jones, 2019). As such we employ a rigorous and multifaceted approach to removing clusters that are homologous to the test set.

The first step is performing HMM-HMM homology searching for each member of CB513 with HHblits (Remmert *et al.*, 2011) using one iteration and an E-value of 10 against the remaining clusters. An accurate means of homology detection, using a high E-value also provides an aggressive sweep to capture any positive matches at the expense of a small number of false hits. One iteration was performed as this was broadly found to return more hits. For removing test set homology, this step acts as a fast single pass to remove a large number of potential homologues.

For the validation set, the same procedure is followed, however, the default E-value ($1\times 10^{-3}$) is used with two iterations. We use these more standard parameters for the validation set as the set is only used for early stopping and not for benchmarking. As such it does not require as aggressive and wide sweeping an approach to removing homologous sequences as is done for the test set. All clusters that are matches to the test and validation sets are then removed.

The remaining clusters are copied and combined to create a single large sequence database which is processed with pFilt (Jones and Swindells, 2002) to mask regions of low amino acid complexity. The test set alignments produced by HHblits are used to construct HMMER (Eddy, 2011) HMMs which are then used to perform HMM-sequence homology searches against the sequence database using hmmsearch. The ‘–max’ flag is used to improve sensitivity and the default e-value is used. All sequences that are positive hits to the test set HMMs, along with their respective clusters, are removed from the remaining pseudo-labelled sequence set.

A secondary and overlapping procedure is also performed. Each member of the test set is mapped to one or more Pfam (El-Gebali *et al.*, 2019) families by pre-existing annotations. These are found by a combination of SIFTS (Dana *et al.*, 2019) and manual searching. From the test set, 17 structures were not found to belong to any Pfam family. For each Pfam family linked to the remaining members of the test set, a list of UniProt sequence IDs is generated. This is extracted from the family’s current UniProt-based Pfam alignment (01-2020) and is used to remove clusters following the same procedure as positive hits from the HMM-sequence search.

In total, these methods remove approximately a quarter of the initial 1.41M clusters, leaving a final 1.08M clusters to construct the final pseudo-labelled training set. While the fear of data leakage remains ever present, we believe that in the absence of structures this process constitutes a rigorous and exhaustive approach to homology removal.

### 2.4 Generating pseudo-labels with PSIPRED

A given cluster can provide a sequence with pseudo-labels by first taking its representative sequence as the target sequence and splitting off the remainder of the cluster alignment. This is treated as if it was the target sequence alignment. Both sequence and alignment are then processed using the standard PSIPRED procedure. The three-class secondary structure labels predicted by PSIPRED V4 (Buchan and Jones, 2019) are then kept along with the target sequence as a single example for the training set. The version of PSIPRED used to generate labels is trained on a set of sequences that are structurally non-homologous with the CB513 test set. This ensures that the pseudo-labels contain no information derived from the test set implicitly through PSIPRED. This procedure is repeated to generate a training set of 1.08M sequences each paired with a sequence of pseudo-labels.

## 3 Results

### 3.1 The prediction of secondary structure from a single sequence

**Fig. 2.**
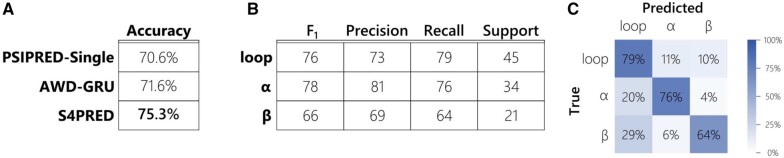
(**A**) Table showing the difference in final accuracy (*Q*_3_ score) between the improved S4PRED, the AWD-GRU benchmark, and the current version of PSIPRED-Single on the CB513 test set. (**B**) Table of classification metrics for the S4PRED model test set predictions. These are shown for each of the three predicted class; *α*-helix, *β*-sheet and loop (or coil). The support is normalized across classes to 100 for clarity—there are a total of 84484 residue predictions in the test set. (**C**) Confusion matrix for the three classes in the S4PRED model test set predictions

**Fig. 3.**
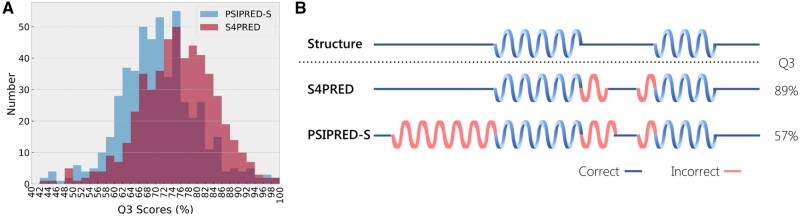
(**A**) Histogram of *Q*_3_ scores on the CB513 test set showing the improved results of S4PRED over PSIPRED-Single (PSIPRED-S). (**B**) Example of S4PRED and PSIPRED-Single secondary structure predictions relative to the true structure for the C terminal domain of pyruvate oxidase and decarboxylase (PDB ID 1POW)

The final model achieves an average test set *Q*_3_ score of 75.3%. This improves the *Q*_3_ of PSIPRED-Single by almost 5% (Fig. 2A), currently being 70.6%. This is clearly seen in Figure 3A, which shows how the distribution of test set *Q*_3_ scores for S4PRED has improved as a whole from PSIPRED-Single scores. In some cases, this has led to a large improvement in prediction accuracy, an example of which is visualized in Figure 3B. Although this represents a significant improvement it is not necessarily a fair comparison as PSIPRED-Single uses a much simpler multi-layer perceptron model (Buchan and Jones, 2019; Jones, 1999).

The most comparable method to date is SPIDER3-Single (Heffernan *et al.*, 2018) which uses a bidirectional LSTM (Hochreiter and Schmidhuber, 1997) trained in a supervised manner. This method predicts secondary structure and other sequence information, like solvent accessibility and torsion angles, from a single sequence. SPIDER3-Single uses one model to make preliminary predictions, which are then concatenated with the original input sequence, to be used as input to a second model that produces the final predictions. It reports a *Q*_3_ score of 72.5%, however, this is on a non-standard test set based on a less stringent definition of homology (Jones, 2019).

To establish an equivalent and informative comparison we provide a second benchmark by training a similar supervised model to SPIDER3-Single which predicts only secondary structure in a standard supervised manner, without a secondary network. This uses the same network architecture as our SSL method but only trains on the labelled sequence dataset. This achieves a *Q*_3_ score of 71.6% on CB513. This is a similar result to that achieved in a recent work (Torrisi *et al.*, 2019), which reported a single-sequence *Q*_3_ score of 69.9% and 71.3% on a validation set with a perceptron model and an LSTM-based model respectively. Although the second benchmark used here does not utilize a secondary prediction network like SPIDER3-Single, it is <1% less performant than SPIDER3-Single’s reported test set performance. Importantly, it provides a direct comparison to S4PRED by using the same model and test set. We use the name AWD-GRU, after the AWD-LSTM variant (Merity *et al.*, 2018) used herein, to refer to this benchmark model. Although they use the same architecture, S4PRED still exceeds the performance of the AWD-GRU benchmark by a difference in *Q*_3_ of almost 4%. Not only is this a large improvement for single-sequence prediction, it directly demonstrates the benefit of the SSL approach.

To more precisely determine the benefit that fine-tuning contributes to this performance gain, we tested a model trained on only pseudo-labelled sequences. This achieves a test *Q*_3_ score of 74.4%. As is expected, this demonstrates that fine-tuning is a functional approach to combining both datasets that markedly improves prediction by ∼1%. Aside from the obvious benefit of learning from real labelled data, we speculate that part of the fine-tuning improvement derives from a softening of class decision boundaries. The model trained on only pseudo-labels has a prediction entropy of 0.325, averaged across classes, residues and sequences. The final model shows a notably higher entropy of 0.548 suggesting that fine-tuning is possibly softening classification probabilities and improving predictions for cases that sit on those boundaries. One clear aspect of S4PRED that should be a focus of future improvement is *β*-strand prediction. Of the three classes, it has the lowest *F*_1_ score by a reasonable margin, 0.66 compared to 0.78 and 0.76 for loop and helix respectively (Fig. 2B). This is likely due to a combination of being the least represented class in the training set and the most difficult class to predict.

As a tool, S4PRED is capable of being run on either a CPU or a GPU. Predicting the secondary structure of a single sequence on a single Threadripper 2950X 3.5 GHz core takes an average of 10.9 s and a median of 9.9 s, for 100 randomly selected sequences from the pseudo-labelled training set. Using a single RTX 2080 Ti GPU the average prediction time is 1.51 s and the median is 1.47 s. If a large number of predictions needs to be made these can be run rapidly in batches. For example, 128 randomly generated sequences of length 500 can be predicted for as a batch in an average of 4.19 s total and a median of 4.22 s, on a GPU.

### 3.2 Predictive performance in the wild

We stress that the testing performed here against CB513 is exactly equivalent to having tested on a set of unseen orphan proteins. When the model predicts the secondary structure for each test sequence, to the model, these sequences are orphans. The model has not been exposed to the test set sequences or their homologues, and in the process of testing only predicts from the individual sequences.

This taken into account we wished to provide a secondary and confirmatory test of model performance on orphan proteins that directly compares against SPIDER3-Single. To do so, we create and test on two further test sets. First, we derived a test set of 23 recently published *de novo* designed proteins (See Supplementary Material S1.1). On this test set S4PRED achieves a *Q*_3_ score of 90.7% and SPIDER3-Single achieves 89.4% (See Table 1). These high *Q*_3_ are unsurprising given *de novo* designed proteins are often designed to have well predicted secondary structure (Marcos and Silva, 2018). However, it is still very encouraging and a sign of generality for S4PRED to have achieved such a high score.

**Table 1.** Showing the *Q*_3_ scores and micro-averaged *F*_1_ scores achieved by S4PRED, SPIDER3-Single and PSIPRED-Single on two test sets; a test set of *de novo* designed proteins (labelled ‘Designed’) and a test set of orphan proteins (labelled ‘Orphans’)

	Q_3_		F_1_	
	Orphans	Designed	Orphans	Designed
**S4PRED**	**75.3%**	**90.7%**	**0.754**	**0.910**
**SPIDER3-Single**	73.3%	89.4%	0.733	0.890
**PSIPRED-Single**	71.1%	86.6%	0.718	0.868

*Note*: Results in bold show the superior performance of S4PRED.

We derived a second test set of 45 recently published orphan proteins (See Supplementary Material S1.2). On this test set S4PRED achieves a *Q*_3_ score of 75.3% and SPIDER3-Single achieves 73.3% (See Table 1). This further confirms that S4PRED is able to accurately predict the secondary structure of orphans and represents a significant improvement in performance.

### 3.3 Data efficiency using the semi-supervised learning approach

Another aspect we wished to investigate was the data efficiency of the SSL approach. We trained the AWD-GRU benchmark model on training sets of different sizes, randomly sampling from the 10 143-sequence real-labelled training set (See Supplementary Material S5). To a good degree, the test set accuracy linearly increases with the logarithm of the real-labelled training set size ($R^{2}=0.92$), as can be seen in Supplementary Figure S1. This trend suggests that the SSL approach simulates having trained on a real sequence dataset that is _**∼**_×7.6 larger. Under the loose assumption that the ratio of PDB structures to labelled training set size stays the same, there would need to be greater than 1.2M structures in the PDB (as compared to the 162 816 entries available as of 04-2020) to achieve the same performance as S4PRED using only real data.

We also looked to estimate the number of sequences that would be required in UniProt (Swiss-Prot and TrEMBL) and other metagenomic sequence resources (Carradec *et al.*, 2018; Mitchell *et al.*, 2020) for a PASS-based model to achieve the current performance of the state-of-the-art homology-based PSIPRED. For each single-sequence method in Figure 1, published since the inception of CATH (Orengo *et al.*, 1997), we find the number of CATH S35 sequence families available the year the method was published. This number serves as a proxy for the number of redundancy-reduced PDB chains that would have been available for generating a dataset. We perform exponential regression between the *Q*_3_ scores and the number of CATH S35 sequence families. The S4PRED result is included however 1.08M is used for the number of families. The resulting regression suggests that 25B non-redundant PDBs or sequence clusters would be required for an S4PRED-like model to reach 84%. We then use the average UniClust30 (2016) sequence cluster depth as a multiplicative factor to estimate the number of raw sequences needed. This provides a soft estimate of a minimum of 160 Billion sequences needed for a method based on PASS, like S4PRED, to achieve similar results to current homology-based models.

### 3.4 Single-sequence prediction in context

In this work we consider single-sequence prediction in the strictest sense. This is a model that, for a single example, provides predictions without using information derived from related sequences or evolutionary information. This is an important distinction because using even a small number of homologous sequences improves prediction by several percentage points (Aydin *et al.*, 2006).

The recently published SPOT-1D (Hanson *et al.*, 2019) provides a clear example of this phenomenon. Hanson and collaborators (Hanson *et al.*, 2019) show *Q*_3_ scores of several homology-based models when predicting with low diversity alignments. The criterion for this low diversity is having *N*_eff_ < 2, a measure of alignment diversity, as provided by HHblits (Remmert *et al.*, 2011). This is reported as *N*_eff_ = 1, however, all values are rounded down to the nearest integer. This is clearly not a single-sequence approach. It is also further evidenced in the reported *Q*_3_ scores. Of the methods reported, Porter 5 (Torrisi *et al.*, 2018, 2019) achieves the highest *Q*_3_ with 78%, followed by SPOT-1D at 77%. Separate to these results, Porter 5 reports a validation set *Q*_3_ of 71.3% when trained on only single sequences without profiles (Torrisi *et al.*, 2019). Ignoring the further potential training set and test set overlap for the values reported in SPOT-1D, this difference in *Q*_3_ clearly demonstrates that using even low diversity alignments is enough to significantly improve predictive performance, over a purely single-sequence approach.

Information from homologous sequences can also improve results by being present in the bias of a trained model. A subtle example of this is in the recent DeepSeqVec model (Heinzinger *et al.*, 2019), which trained an unsupervised neural network to produce learned representations of individual sequences from UniRef50 (Suzek *et al.*, 2015). The unsupervised model is subsequently used to generate features which are used to train a second model that predicts secondary structure. This second model achieves a *Q*_3_ score of 76.9% on CB513 (Heinzinger *et al.*, 2019). Although this two model approach is providing secondary structure predictions for individual sequences, it is not a single-sequence method because the unsupervised model has access to implicit evolutionary information for both the training set and test set sequences. This is partly due to being improperly validated, a split was not performed between the training and test sets. With no split the model is able to learn relationships between test set and training set sequences. It is also due to the training objective of the underlying ELMo language model (Peters *et al.*, 2018). The model is able to learn relationships between homologous sequences in a shared latent space, especially given that residue representations are optimized by trying to predict what residue is likely to be found at each position in a given input sequence.

Even if the model uses a small amount of evolutionary information, it still precludes it from being a single-sequence method. The predictions from such a model still benefit from evolutionary information. This not only highlights the difficulty in developing accurate methods that are strictly single-sequence, it also highlights how achieving a *Q*_3_ score of 75.3% with S4PRED represents a step up in performance for single-sequence methods.

## 4 Discussion

Secondary structure prediction from the typical homology-based perspective has improved year-on-year and published *Q*_3_ scores are beginning to rise above 85%. It is non-trivial to disentangle the exact relationship between the amount of data available and model performance but the different versions of PSIPRED provide a valuable insight. From an architecture and training perspective, the current version (Buchan and Jones, 2019) (V4) remains mostly similar to the original first published model (Jones, 1999), yet the current version is a state-of-the-art model under strict testing criteria (Buchan and Jones, 2019). The primary difference between versions is the much larger available pool of training examples. This suggests strongly that the primary bottleneck on performance has been data availability.

Looking to single-sequence prediction, it stands to reason that methods have improved relatively little over time. Data availability, or more generally the amount of information available to a classifier, appears to be a driving force in performance, and by their very nature single-sequence methods have much less available information. This is likely applicable across many orphan sequence modelling tasks, not just secondary structure prediction (Greener *et al.*, 2019; Perdigão *et al.*, 2015). In this work, we developed and applied the PASS framework to directly tackle this issue of data availability. This led to the development of S4PRED which, in achieving a leap in single-sequence performance, stands as an exemplar to the effectiveness of the PASS approach. PASS, and S4PRED, leverages a semi-supervised approach to provide a neural network classifier with information from over a million sequences. Not only is this successful, it is also a conceptually simple approach. A homology-based method (in this case PSIPRED) is used to generate accurate labels for unlabelled examples. The new example and label pairs are then combined with real-labelled data and used to train a single-sequence-based predictor.

S4PRED has achieved significant progress in improving single-sequence secondary structure prediction, but there is still much work to be done. There remains an 8–9% performance gap between S4PRED and current state-of-the-art homology-based methods (Yang *et al.*, 2018). Given the importance of data availability, an immediate question that arises is whether the best approach to closing the gap is to simply wait for larger sequence databases to be available in the near future. To an extent, this appears to be a feasible approach. The number of entries in UniProt grows every year (UniProt-Consortium, 2019) and a massive amount of data is available from clustered metagenomic sequences in databases like the BFD (Steinegger *et al.*, 2019; Steinegger and Söding, 2018).

It is likely that increasing the training set every year will improve performance but to what extent is unknown and the computational cost will correspondingly increase. An increase in training set size will also be dictated by an increase in the number of new families in a database (a sequence cluster being a proxy for a family) and not the number of new sequences. Our estimations suggest that 160 Billion sequences would be required to match homology levels of performance with a PASS method. Given the speed at which sequence databases are growing (Steinegger *et al.*, 2019; UniProt-Consortium, 2019) this is not unreasonable, but unlikely to be within reach in the near future. Instead, a focus on methodological improvements stands to yield the best results.

Looking forward, it is always difficult to speculate what specific methods will result in further improvements. Continuing from the perspective of secondary structure prediction, the field has, in recent years, focused on developing larger and more complex neural networks (Yang *et al.*, 2018). There is certainly a benefit to this approach. Prototyping tends to be quick so any improvements found can be shared with the scientific community quickly. Unfortunately, there is limited novelty in this overall approach and, most importantly, the results of applying the PASS framework suggest that there are only small gains to be had. Waiting for databases to grow in size, and for the development of more complex network architectures, is unlikely to be the answer. Instead, focusing on developing methods that provide pre-existing models with more prediction-relevant information will likely result in the most significant progress.

The most obvious approach to this kind of development is to explore further techniques from semi-supervised learning. Methods like data augmentation, that have shown success with image data (Berthelot *et al.*, 2019; Sohn *et al.*, 2020), would be ideal in getting the most out of the data that is available. Unfortunately, it is non-trivial to augment biological sequences even when the structure or function is known which makes data augmentation a difficult approach to pursue (Kandathil *et al.*, 2019a). That being said, homologues of a given sequence in the training set can loosely be viewed as biologically valid augmentations of the original target sequence. From this perspective, including multiple pseudo-labelled sequences from each cluster as separate examples, instead of the current method which only includes a single target sequence from each cluster, could be viewed as a proxy for data augmentation. Another approach to improving results may be to train models like S4PRED to predict the class probabilities outputted by the label-providing homology model, instead of predicting the hard class assignments, in a manner similar to Knowledge Distillation (Hinton *et al.*, 2015). Alternatively, S4PRED could be limited to learning only labels predicted by PSIPRED with a high degree of confidence. A more general method like MixUp (Zhang *et al.*, 2018), that is application domain agnostic, might also improve classification by improving the classifiers overall generalizability. Suffice it to say, the semi-supervised approach of PASS brings with it a variety of potential ways to improve performance by directly providing more information to the classifier.

Given the success of S4PRED, PASS provides a simple blueprint from which further methods can be developed for modelling orphan sequences. An obvious first step with protein sequences is looking to predict other residue level labels like torsion angle prediction (Heffernan *et al.*, 2018), or even extending to the difficult task of protein contact prediction (Kandathil *et al.*, 2019b). PASS could also be applied to other biological sequences, such as in the prediction of RNA annotations (Hanumanthappa *et al.*, 2021). Extending PASS to other prediction tasks in the future will also likely be aided by recent efforts to consolidate databases of sequences with pre-calculated predictions of various attributes from a range of tools. One such example being the residue-level predictions provided in DescribePROT (Zhao *et al.*, 2021). As more of the protein universe is discovered the need for methods that are independent of homology only grows. Methods like S4PRED will hopefully come to represent a growing response to this need, the PASS framework providing a path forward. With this in mind we provide S4PRED as an open source tool and as an option on the PSIPRED web service. We also make the 1.08M example pseudo-labelled training set publicly available from our web service as a flat file for further research and investigation.

## Supplementary Material

btab491_Supplementary_Data
