# Supplementary material for: Increasing the accuracy of single sequence prediction methods using a deep semi-supervised learning framework
Source: Bioinformatics. 2021 Jul 2;37(21):3744–51. doi: 10.1093/bioinformatics/btab491 (PMC8570780; doi:10.1093/bioinformatics/btab491)
Supplement: btab491_Supplementary_Data [file btab491_supplementary_data.pdf]

Increasing the Accuracy of Single Sequence Prediction Methods Using a Deep Semi-Supervised Learning Framework: Supplementary Materials

Lewis Moffat, David T. Jones

March 2021

Additional Methods

S1 Additional Test Sets

S4PRED and SPIDER3-Single are tested against two additional test sets: a set of recently released orphan proteins and recently released *de novo* designed proteins. SPIDER3-Single was downloaded from https://sparks-lab.org/downloads/ and run locally.

S1.1 *De Novo* Designed Protein Test Set

The *de novo* designed protein test set constitutes 23 protein chains released in the PDB (Burley *et al.*, 2019) from January 2020 till February 2021. This release range precludes the proteins in this set from having been included in the S4PRED and the SPIDER3-single training sets. A list of PDB IDs for protein structures in this date range with the 'DE NOVO PROTEIN' classification were first redundancy reduced using the PISCES server (Wang and Dunbrack Jr, 2003) at the chain level (with a 70% identity threshold and otherwise default parameters). The remaining PDB chains are manually inspected to remove non-*de novo* designed protein chains (e.g. PDB ID: 6YWC, which contains immunoglobulin chains in complex with a *de novo* designed protein). This final set is then used to test both S4PRED and SPIDER3-Single. This is a strong test of generality as it is an exactly equivalent scenario to testing both models on designed sequences that have yet to be structurally characterized i.e. sequences and structures not a part of the training and validation sets.

S1.2 Orphan Protein Test Set

The orphan protein test set constitutes 45 protein chains release in the same date range as the *de novo* designed protein test set. The set was constructed

by first acquiring a list of PDB IDs released in that date range. This set is then redundancy reduced at 30% identity threshold using the PISCES server (Wang and Dunbrack Jr, 2003) (otherwise default parameters). Each protein chain in the remaining test set is then run against the Uniclust30 database (Jan-2021 release) using `HHblits` (Remmert *et al.*, 2012) with an E-value of 1^{-3}. The sequence identity threshold was set to 100% and the `-diff` flag was set to `inf`. This is intended to be very permissive and count even sequences with one deletion as a distinct homologue.

Sequences which returned an alignment of 9 or fewer homologues are considered sequence orphans. This is not only a measure of being an orphan but also precludes any homologues of the sequence having be used in S4PRED's psuedo-labelled training set. The filtering results in a final set of 45 orphan protein sequences.

S2 Neural Network Model Architecture

We use a state-of-the-art recurrent neural network (RNN) from the language modelling domain as a classification model. More specifically we adapt the AWD-LSTM (Merity *et al.*, 2018) for secondary structure prediction. The first portion of our model takes a sequence of amino acids encoded as integers and replaces them with corresponding 128-d embeddings that are learned during training and are initialized from $\mathcal{N}(0, 1)$. During training a 10% dropout is applied to the embeddings.

The embeddings are fed into a bidirectional gated recurrent unit (GRU) (Cho *et al.*, 2014) model with 1024 hidden dimensions in each direction. Here the model differs from the AWD-LSTM which utilizes a long short term memory (LSTM) model with DropConnect (Wan *et al.*, 2013) applied to the hidden-to-hidden weight matrices. Our model does the same but utilizes a GRU which we refer to as an AWD-GRU. Unless specified, the weight dropping is set to 50% during training. This model utilizes three layers of AWD-GRUs with 10% dropout applied between each layer during training.

The output of the final recurrent layer is a 2048-d vector at each time step. This is fed into a final linear layer with a log softmax operation to produce the 3-class probabilities at each residue position. These are then used to calculate a negative log likelihood loss using the corresponding one-hot encoded labels. Unlike the original AWD-LSTM we use another popular stochastic gradient descent (SGD) variant, Adam (Kingma and Ba, 2015), as an optimizer to minimize the loss and train model parameters.

S3 S4PRED training with pseudo-labelled data

The first stage in training the S4PRED model is training on the 1.08M pseudo-labelled sequences. For optimization the Adam beta terms are set to β_1,β_2 = $\{0.9, 0.999\}$ with an initial learning rate of 1×10^{-4} and a mini-batch size of 256 (See S2 and S3 for further architecture and implementation details). We also perform gradient clipping with a maximum norm of 0.25. To utilize a

batch size of greater than 1 all batches are padded on the fly to the length of the longest sequence in a given batch. The padding symbol has a corresponding embedding and the loss is masked at positions that are padded. Training occurs for up to 10 epochs which typically takes between 48 to 72 hours in total. The performance on the validation set is tested every 100 batches and it is used to perform early stopping.

S4 Fine-tuning with labelled data

We adapt the methodology presented by Devlin and collaborators (Devlin *et al.*, 2019) for S4PRED by taking the model trained on pseudo-labelled sequences and performing 1 epoch of training on the 10K labelled sequences. Unlike their method, however, we do not need an additional output layer, having already trained on the semi-supervised secondary structure prediction objective with the psuedo-labelled sequences. For fine-tuning, the batch size is lowered to 32 and the weight drop is set to 0%. All other hyper-parameters are kept the same and the Adam optimizer is reset. The final model is a an ensemble of 5 models fine-tuned with different random seeds, all starting from the same model. Using an ensemble improves prediction by $\sim 0.1\%$.

S5 Performance benchmarking

Two methods are used to benchmark the results of S4PRED. The first method is the original PSIPRED-Single. Its predictions are generated using the pipeline included with PSIPRED V4. PSIPRED-Single achieves a Q_3 score of 70.6% on CB513. The AWD-GRU model is the second model used for benchmarking. It is trained with the same model architecture and hyper-parameters as S4PRED when it is being trained on the psuedo-labelled set before fine-tuning. However, it only trains on the 10143-sequence set with real labels. This achieves a Q_3 score of 71.6% also on CB513.

The data efficiency of the S4PRED method was investigated to estimate the value of training with pseudo-labelled data. This was done by training five versions of the AWD-GRU model, each with a different random seed, on different sized subsets of the 10143 real labelled data. Models were trained with 100, 500, 1000, 2500, 5000, 7500, & 10143 examples (a total of 35 models). Each model is tested against CB513 and a linear regression model is fit between the logarithm of the number of points and model Q_3 score ($R^2 = 0.92$). This is visualized in Figure S1. By the linear model, a Q_3 score of 75.3% would require 77K real labelled sequences in the dataset.

S6 Software implementation

All analysis was performed using Python and all neural network models were built and trained using Pytorch (Paszke *et al.*, 2019). During training, all models used mixed precision which was implemented using the NVIDIA Apex package with the `-O2` flag. This was found to improve training speeds with a negligible

effect on results. Individual models were trained on a single compute cluster node using a single NVIDIA V100 32GB GPU. Upon publication, the S4PRED model and AWD-GRU model with their weights will be released as open source software on the PSIPRED GitHub repository (https://github.com/psipred/) along with documentation. It will also be provided as a part of the PSIPRED web service (http://bioinf.cs.ucl.ac.uk/psipred/).

S7 Homology bias in single residue mutants

Homology based methods like PSIPRED that use MSAs are limited compared to single-sequence approaches in that their predictions for a given sequence will have a bias towards a family "average" (Kandathil *et al.*, 2019). In avoiding this bias, single-sequence methods have the potential to better model changes in secondary structure across a family. We believe this to be a valuable future avenue for research, and we provide an illustrative example of this phenomenon here in Figure 3.

We take three structures, each a member of a large family, and for each we mutate a residue in the center of a randomly chosen helix to proline. We would typically expect for this mutation to disrupt the helix structure. As such, it would be expected that both S4PRED and PSIPRED would reflect this in predicting a coil (loop) region for the mutant where they previously predicted helix for the native sequence. However, as can be seen in Figure 3, S4PRED predicts a change in the structure but PSIPRED retains its prediction of a helix. Even a double mutation of proline at the sites shown does not change the PSIPRED prediction of a helix (not included in the figure for clarity). This provides a simplistic but clear example of the bias that can be present in homology based models towards the average prediction of a family. It additionally demonstrates how single-sequence models may have the potential to ameliorate this bias.

Figures

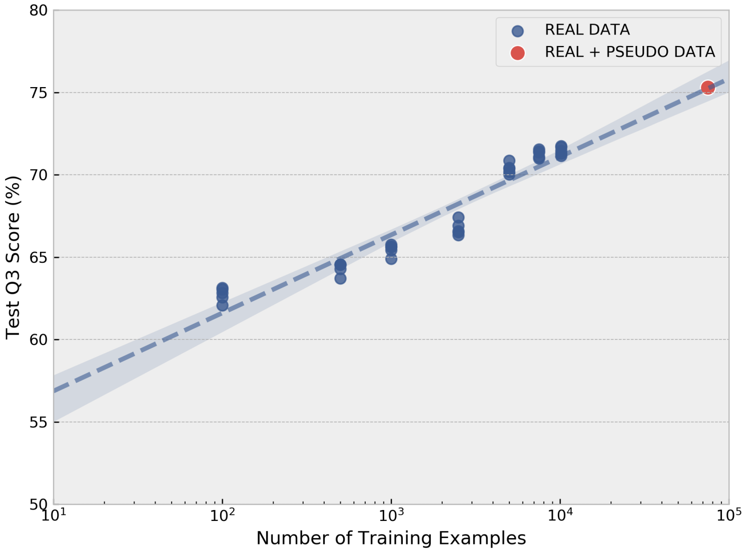

Figure 1: Scatter plot comparing the logarithm of the number of data points compared to trained model accuracy with real labelled sequences. A dashed linear trend line is included. The S4PRED model using real and psuedo-labelled data (75.3%) is included as a single point for comparison.

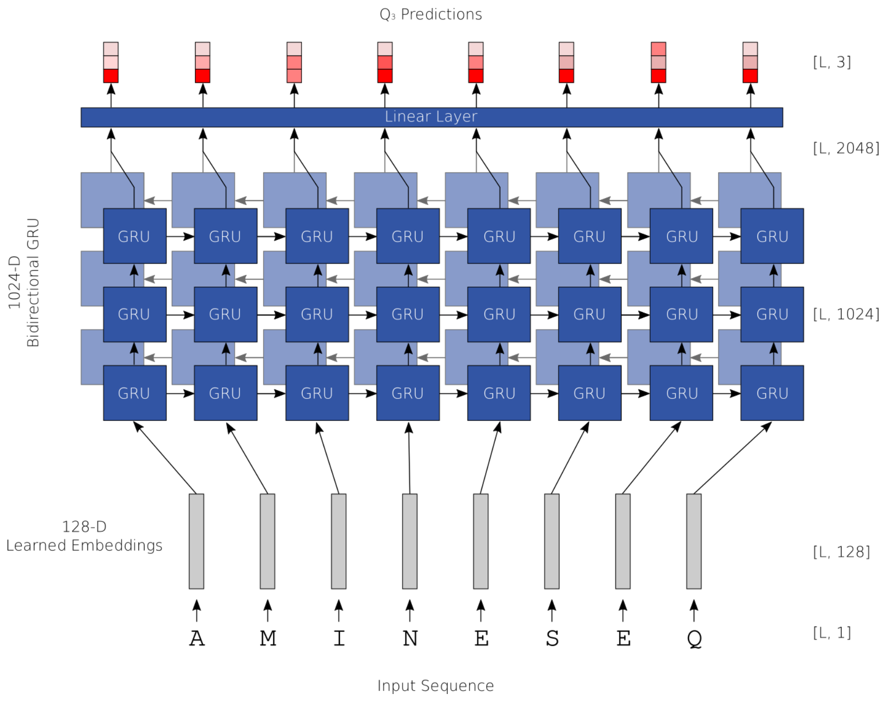

Figure 2: The architecture of the S4PRED model when being used during inference. The amino acid sequence, `AMINESEQ`, is used as an illustrative example of an input sequence. The dimensions of the example as it progresses through the network are shown on the right, where L represents the sequence length ($L = 9$ in the case of `AMINESEQ`). Note that due to the network being bidirectional the input is 1024-D for the forward and backwards models. The concatenated output of both leads to the 2048-D tensor.

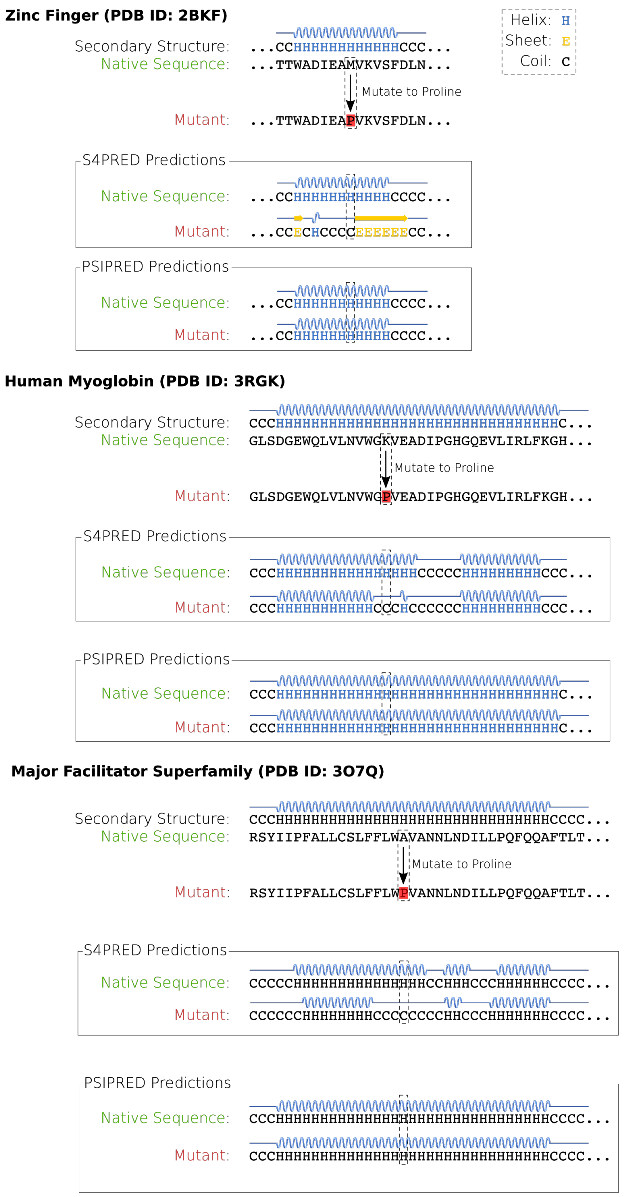

Figure 3: Diagram demonstrating the effect of performing single residue mutations, in three different structures, on PSIPRED and S4PRED secondary structure predictions. For each example, a residue in the center of a randomly chosen helix is mutated to proline. Typically, this is expected to destabilise the helix. This is evident in that the S4PRED predictions reflect a change in predicted structure from the native sequence whereas the PSIPRED predictions do not. This provides an illustrative view of how a homology approach can be biased towards the average of the family.

References

Burley, S. K. *et al.* (2019). Rcsb protein data bank: biological macromolecular structures enabling research and education in fundamental biology, biomedicine, biotechnology and energy. *Nucleic acids research*, **47**(D1), D464–D474.

Cho, K. *et al.* (2014). Learning phrase representations using rnn encoder–decoder for statistical machine translation. In *Proceedings of the 2014 Conference on Empirical Methods in Natural Language Processing (EMNLP)*, pages 1724–1734.

Devlin, J. *et al.* (2019). Bert: Pre-training of deep bidirectional transformers for language understanding. In *Proceedings of the 2019 Conference of the North American Chapter of the Association for Computational Linguistics*, pages 4171–4186.

Kandathil, S. M. *et al.* (2019). Recent developments in deep learning applied to protein structure prediction. *Proteins: Structure, Function, and Bioinformatics*, **87**(12), 1179–1189.

Kingma, D. P. and Ba, J. (2015). Adam: A method for stochastic optimization. In Y. Bengio and Y. LeCun, editors, *3rd International Conference on Learning Representations, ICLR 2015, San Diego, CA, USA, May 7-9, 2015, Conference Track Proceedings*.

Merity, S. *et al.* (2018). Regularizing and optimizing LSTM language models. In *6th International Conference on Learning Representations, ICLR 2018, Vancouver, BC, Canada, April 30 - May 3, 2018, Conference Track Proceedings*. OpenReview.net.

Paszke, A. *et al.* (2019). Pytorch: An imperative style, high-performance deep learning library. In *Advances in Neural Information Processing Systems*, pages 8024–8035.

Remmert, M. *et al.* (2012). Hhblits: lightning-fast iterative protein sequence searching by hmm-hmm alignment. *Nature methods*, **9**(2), 173.

Wan, L. *et al.* (2013). Regularization of neural networks using dropconnect. In *30th International Conference on Machine Learning, ICML 2013*, pages 2095–2103. International Machine Learning Society (IMLS).

Wang, G. and Dunbrack Jr, R. L. (2003). Pisces: a protein sequence culling server. *Bioinformatics*, **19**(12), 1589–1591.
